# Cross-protectivity of henipavirus soluble glycoprotein in an *in vivo* model of Nipah virus disease

**DOI:** 10.3389/fimmu.2025.1517244

**Published:** 2025-02-26

**Authors:** Stephen Findlay-Wilson, Nazia Thakur, Lucy Crossley, Linda Easterbrook, Francisco J. Salguero, Ines Ruedas-Torres, Susan Fotheringham, Emma Kennedy, Dalan Bailey, Stuart Dowall

**Affiliations:** ^1^ Specialised Microbiology and Laboratories, United Kingdom Health Security Agency (UKHSA), Salisbury, Wiltshire, United Kingdom; ^2^ Viral Glycoproteins, The Pirbright Institute, Woking, United Kingdom; ^3^ Nuffield Department of Medicine, University of Oxford, Oxford, United Kingdom

**Keywords:** Nipah virus, vaccine, glycoprotein, adjuvant, cross-protectivity

## Abstract

**Introduction:**

Nipah virus (NiV) is one of a group of highly pathogenic viruses classified within the Henipavirus genus. Since 2012 at least 11 new henipa-like viruses have been identified, including from new locations and reservoir hosts; the pathogenicity of these new viruses has yet to be determined, but two of them have been associated with morbidity, including fatalities.

**Methods:**

The efficacy and cross-reactivity of two vaccine candidates derived from the soluble glycoproteins of both NiV and Hendra virus (HeV) was evaluated in our recently established hamster model.

**Results:**

Both vaccine preparations resulted in strong humoral responses against NiV antigenic targets, demonstrating cross-reactive immunity. Efficacy was determined through challenge of hamsters with NiV Malaysian (NiV-M) strain. 100% of the hamsters survived a lethal challenge dose after prime/boost immunisation with glycoproteins derived from both NiV and HeV in the presence of adjuvant, with clinical signs and pathology being significantly reduced in immunised animals.

**Discussion:**

This is first time the NiV and HeV soluble glycoproteins have been compared in the NiV-M hamster challenge model in the presence of Alhydrogel and AddaVax, providing evidence that glycoproteins from closely related henipavirus species can provide cross-protectivity against infection from alternate henipaviruses, supporting the potential of an effective pan-henipavirus vaccine for use in a frontline outbreak response.

## 1 Introduction

Nipah virus (NiV) is a non-segmented, negative sense, single stranded RNA virus taxonomically classified within the Henipavirus genus (family *Paramyxoviridae*), capable of causing severe disease in humans and high levels of economic loss (1). Fruit bats (family *Pteropodidae*) are the principal reservoirs for henipaviruses and are widely distributed in some of the most populated areas of the world, many of which are classified as lower- and middle-income countries (LMIC), enhancing the chances of the establishment of an undetected outbreak. Henipavirus-like genetic material and antibodies have been detected in Australia, Bangladesh, Cambodia, China, East Timor, Ghana, India, Indonesia, Malaysia, Madagascar, Papua New Guinea, Vietnam, Singapore, Thailand and throughout South America (1, 2). NiV was first discovered in an outbreak affecting domestic pigs and people in Kampung Sungai Nipah Village, in Perak state, Malaysia in 1998, which caused 265 cases of clinical encephalitis and 105 fatalities - a case fatality rate (CFR) of 40% - and over 1 million pigs were slaughtered to contain the spread of the virus (3–5). The ‘Malaysian’ strain of NiV (NiV-M) is associated with high levels of respiratory and neurologic illness and has been implicated in two outbreaks in Malaysia and Singapore (4). In 2001, a new strain of NiV was identified during an outbreak in a village in Meherpur District, Bangladesh (NiV-B) (4). This strain has a 91.8% genomic similarity with NiV-M and has been associated with almost annual outbreaks in Bangladesh and India, with a CFR recorded of up to 100% (6, 7).

Since 2012 various new members of the Henipavirus genus (*H. cedarense, H. ghanaense and H. angavokelyense*) and henipa-like viruses (*Parahenipavirus mojiangense, P. wenzhouense, P. gamakense, P. daeryongense, P. soricis, P. meliandouense, P. winnikense, P. jingmenense, P. chodsigoae, P. crocidurae* and *P. langyaense*) have been discovered, either through isolation, or based on sequence analysis (6, 8–10). These discoveries have increased the known distribution range of these viruses – *P. daeryongense* and *P. gamakense* in Korea, *P. winnikense* in Belgium and Peixe-Boi virus (PBV) in Brazil (the latter of particular interest being the first sequence confirmation of henipa-like viruses in the Americas, significantly expanding the range of henipavirus distribution (6, 11). In addition, the number of known mammalian species capable of acting as reservoir hosts has expanded: *P. daeryongense, P. gamakense, P. winnikense, P. langyaense* and *P. meliandouense* were identified from sequences obtained from shrews, *P. mojiangense* from the cave rat, *P. wenzhouense* from the striped field mouse, and PBV from Brazilian opossums. Although little is known regarding the pathogenicity of these viruses, *P. langyaense* was detected during a surveillance study in 35 patients exhibiting acute fever and has been associated with respiratory symptoms including fever, cough and fatigue (12). Similarly, *P. mojiangense* has been linked with human disease including the death of three minors from severe pneumonia (13).

Members of the Henipavirus genus, including Hendra virus (HeV), are included on the priority pathogens lists for the World Health Organisation (WHO) R&D Blueprint (14), the Coalition for Epidemic Preparedness Innovations (CEPI) and the UK Vaccine Network (15). However, there are no Food and Drug Administration (FDA) henipavirus vaccines approved for clinical use, although there are four vaccine candidates at phase I clinical trials (16, 17). One of the major factors for this lack of progression is the unpredictability of human outbreaks on which to test these vaccines. As such, a lot of work has gone into the development of well-characterised *in vivo* models of henipavirus infection for testing the efficacy of vaccine candidates to satisfy the US Food and Drug Association’s Animal Rule (18). The Golden Syrian hamster (*Mesocricetus auratus*) is one of the most utilised of these models due to its small size, ease of handling, and ability to replicate many of the clinical signs associated with human infection (19–23).

In a previous study, we used the Syrian hamster as a model for NiV-M infection, comparing the intranasal (i.n.) challenge route with intraperitoneal (i.p.) delivery, and for dose-ranging and investigative studies into the kinetics of infection. Analysis of the data revealed a dose-dependent effect with higher challenge titres leading to more respiratory associated sequalae, whereas lower challenge titres led to more neurological-based signs (24).

With the escalation in the number of related species of henipavirus being discovered in new geographical areas and from distinct reservoir species, we applied our recently established hamster model of NiV-M strain disease to evaluate the protective effects of soluble glycoprotein (sG) vaccine candidates. These immunogens, derived from both NiV-M and HeV glycoproteins, are truncated glycoproteins that have had the transmembrane and cytoplasmic tail removed enabling secretion and purification. Based on a similar technology used by Zoetis in the production of the HeV vaccine (Equivac HeV) licensed for use in horses (25–27), these vaccines are readily available and relatively inexpensive to produce. Immunising with both NiV-M and HeVsG in the Syrian hamster model provides further evidence to the cross-protectivity of the vaccines after challenge with NiV-M strain, leading to greater insights into the potential of producing an effective pan anti-henipavirus vaccine.

## 2 Materials and methods

### 2.1 Animals

Golden Syrian hamsters were obtained from a UK Home Office accredited supplier (Envigo, UK). Thirty hamsters at ≥ six weeks of age were divided into five groups (n = 6 per group) with an equal allocation of male and female animals per group. After a period of acclimatisation, the animals were ID chipped, weighed and temperatures taken. Day one pre-immunisation, baseline blood samples (100µL) were taken for serum separation and subsequent antibody analysis. Hamsters were immunised intramuscularly (i.m.) with 100 µL of vaccine across two sites using a prime-boost regimen, with three-weeks between each vaccination and subsequent challenge. Further blood samples for antibody analysis were taken the day before boost-immunisation and pre-challenge. Hamsters were challenged intraperitoneally (i.p.) with 100 TCID_50_ NiV-M, a dose that has previously been demonstrated to replicate the respiratory and neurological clinical signs of disease observed in humans (24). Food and water were available *ad libitum*, with environmental enrichment included in cages. All animal experimental protocols were approved by ethical review at the UK Health Security Agency by the Animal Welfare and Ethical Review Body (AWERB) and performed under Home Office project licence: PP3877532.

### 2.2 Vaccines and adjuvants

Recombinant protein NiV-M and HeVsG vaccines were produced by cloning the ectodomain of the NiV-M (GenBank AY816745.1) or HeV (GenBank AAC83193.2) G sequences into the pHLSec expression vector and expressed in Expi293 cells using PEI40K transfection reagent (Polysciences, USA) with additives (300mM valproic acid, 500mM sodium propionate, 2.4M glucose). Supernatants were harvested 4 days post-transfection and purified using HisTrapHP columns (Cytiva, USA). Proteins were then desalted using Zeba Spin Desalting Columns (Fisher Scientific, UK) and concentrated using Amicon Ultra 15 columns (Merck Millipore, USA) before quantification using a Nanodrop and Pierce**™** BCA Protein assay (Thermo Scientific, USA). These proteins were administered at a concentration of 5 µg/hamster, based on similar studies (28–31) in conjunction with two commercially-available adjuvants: AddaVax, a squalene-based oil-in-water nano-emulsion with a formulation similar to that of MF59^®^ and Alhydrogel (referred to as Alum), an aluminium hydroxide wet gel suspension (InvivoGen, France).

### 2.3 Virus and challenge

NiV-M (GenBank no. AF212302) was kindly provided by the Special Pathogens Branch of the Centers for Disease Control and Prevention, Atlanta, USA. Virus was propagated and titrated on VeroE6 cells (European Collection of Cell Cultures, UK) grown using Dulbecco’s minimal essential medium (DMEM; Gibco, UK) supplemented with 2% foetal bovine serum (Gibco, UK) at 37**°**C. All infectious work was performed in Containment Level (CL) 4 facilities at UKHSA, Porton Down.

Virus was diluted in sterile phosphate buffered saline (Gibco, UK) to achieve the challenge dose of 100 TCID_50_. For challenge, the virus was injected via the i.p. route in a volume of 200 µL. Challenge was given under isoflurane sedation and animals monitored until a full recovery from sedation was observed.

### 2.4 Clinical observations

Throughout the study, clinical signs were recorded at least twice daily by experienced husbandry and animal welfare staff (this was increased to four times a day if hamsters started showing moderate signs of disease). At equivalent times each day (07:00-09:00) animals were weighed and had temperatures recorded via an implantable ID/temperature chip (idENTICHIP with Bio-Thermal, Identichip, UK). Clinical signs of disease were assigned a score based on the following criteria: 0, healthy; 1, behavioural change, eyes shut; 2, ruffled fur; 3, wasp-waisted, arched back, dehydrated; 5, laboured breathing; 8, ataxia, neurological signs and paralysis; and 10, immobility (24). For analysis, a cumulative score combining all observed signs was then assigned for each animal at that time point.

### 2.5 Necropsy procedures

Hamsters were anaesthetised with isoflurane followed by an overdose of sodium pentobarbital at the scheduled end of the study (14 days post-challenge) or upon meeting humane clinical endpoint criteria. At necropsy a sample of blood was collected into animal blood RNAprotect tubes (Cat. No. 76544, Qiagen, UK) and a sample of brain, liver, lung, and spleen into dry tubes. These were stored at -80**°**C until preparation for viral RNA analysis. The remainder of the brain, liver, lung and spleen was collected into histology pots containing 10% neutral-buffered formalin (NBF) for fixation by immersion and further histopathological analysis.

### 2.6 Quantification of IgG antibody response by indirect ELISA

ELISA were performed as described previously (32). 96-well plates (Nunc MAXIsorp, Thermo Fisher Scientific) were coated with 100 ng/well of recombinant NiV-M sG protein diluted in 100 μL 0.06 M carbonate/bicarbonate buffer pH 9.6 (Merck Millipore, USA) and incubated overnight. Plates were blocked with 5% skimmed milk in PBS at 37**°**C for 2 h. After removal of block buffer, hamster serum samples were diluted in PBS two-fold starting at 1:400 and added to the plate in 2.5% milk in PBS-Tween (0.1%) (PBST) and incubated for 1 h at 37**°**C. Plates were washed 3 times with PBST before the addition of horseradish-peroxidase (HRP)-conjugated anti-Syrian hamster IgG polyclonal antibodies (107-005-142, Jackson ImmunoResearch, USA) diluted at 1:10,000. Plates were incubated for 1 h at 37**°**C and then washed 3 times with PBS-T. TMB solution (Merck) was added and plates incubated for 3 min at room temperature in the dark before the addition of an equivalent volume of 1M sulphuric acid stop solution. Absorbance was read at 450 nm using the Glo-Max^®^ Multi+ Detection System (Promega, UK). Antibody end-point titres were calculated as the reciprocal of the highest dilution at which the OD value was greater than the cut-off value (mean + 3SD of a negative serum).

### 2.7 Pseudovirus-based assays to measure neutralising antibody responses

NiV-M pseudoviruses (NiV-Mpp), produced as previously described, were used to measure neutralising antibody responses, as these had been shown to be an effective and safer alternative to live NiV neutralisation assays performed in CL4 facilities (26, 27, 32, 33). Briefly, HEK293T cells were plated at a density of 2 × 10^6^ cells per 10 cm^2^ dish. The following day, the cells were transfected with 1 µg each of pcDNA3.1 plasmid expressing NiV-M G and F along with 1 µg p8.91 (encoding for HIV-1 gag-pol) and 1.5 µg CSFLW (the firefly luciferase reporter-expressing lentivirus-backbone) and 20 µL PEI (Sigma-Aldrich). Virus was harvested at 24 and 48 h post transfection and titrated 10-fold on BHK-21 cells to measure infectivity, based on luciferase expression. Sera from vaccinated hamsters were diluted 1:40 in triplicate and titrated 4-fold. A fixed volume of NiV-Mpp equivalent to 1 × 10^5^ signal luciferase units was added and incubated at 37°C for 1 h then overlayed with BHK-21 target cells (2 × 10^4^/100 µL). After 72 h, firefly luciferase activity was measured using the Luciferase Assay System substrate (Promega, UK) as per manufacturer’s instructions on a Glo-Max^®^ Multi+ Detection System (Promega, UK). Serum neutralisation titres were calculated by interpolating the dilution at which 80% inhibition of luciferase values (IC_80_) was detected, compared to no sera controls.

### 2.8 Quantification of viral loads by RT-qPCR preparation

Tissue samples for viral RNA analysis were weighed, resuspended in 1.5 mL PBS and homogenised through a 400 μm mesh in a Netwell plate (Corning, UK). 200 μL of tissue homogenate or blood was transferred to 600 μL RLT buffer (Qiagen, UK), mixed by inversion, and after at least 10 minutes 600 μL 70% molecular grade isopropanol (Fisher Scientific, UK) was added to each sample. Samples were then transferred from the CL-4 suite to a CL-3 laboratory where contents were transferred to new tubes for RNA extraction outside of containment. Tissues were further homogenised through a QIAshredder (Qiagen, UK) at 16,000 x g for 2 minutes and RNA extracted by KingFisher Flex automatic extraction using the BioSprint 96 one-for-all veterinary kit (Indical, UK) as per manufacturer’s instructions and eluting into 100 μL AVE buffer (Indical, UK). Samples were analysed by RT-PCR using the TaqMan Fast Virus 1-Step Master Mix RT-PCR kit (ThermoFisher, UK) using the fast-cycling mode and primers/probes targeting the NP gene of NiV-M (NCBI Reference Sequence: NC_002728.1) adapted from Guillaume et al., 2004 (30, 34) Primer/probe sequences: forward (NP1209) 5’-GCAAGAGAGTAATGTTCAGGCTAGAG-3’, reverse (NP1314) 5’-CTGTTCTATAGGTTCTTCCCCTTCAT-3’, fluorescent probe (NP1248) 6FAM 5’-TGCAGGAGGTGTGCTCATTGGAGG-3’ BHQ1.Quantification of viral load was determined using a 10-fold serial dilution of NiV N gene *in vitro* transcript [2.0x10^6^ to 2.0x10^0^ copies µL-1] (Integrated DNA Technologies, UK).

### 2.9 Histopathology and *in-situ* hybridisation

Tissue samples, including lung, brain, liver and spleen, were fixed in 10% NBF for 3 weeks and then routinely processed and embedded into paraffin wax. Tissue blocks were cut into 4 µm sections and routinely stained with haematoxylin and eosin (H&E). Slides were digitalised using a Hamamatsu S360 digital slide scanner (Hamamatsu Photonics K.K., Shizuoka, Japan) and examined with the ndp.view2 software (Hamamatsu Photonics K.K., v2.8.24).

The severity of histopathological lesions in all organs was recorded using a semi-quantitative scoring system. The parameters evaluated in each organ were: broncho-interstitial pneumonia in the lung; the presence of meningitis and perivascular cuffing in the brain; the presence of inflammatory infiltrates in the liver and the presence of polymorphonuclear and mononuclear cell infiltrates and lymphoid depletion in the spleen. For each parameter the following scores were applied 0 = within normal limits; 1 = minimal; 2 = mild; 3 = moderate and 4 = marked/severe. The cumulative sum of all the scores was calculated to give a histopathology score for each individual animal.

Further 4 µm sections were stained using the *in-situ* hybridisation (ISH) RNAscope technique to identify NiV RNA. Briefly, slides were pre-treated with hydrogen peroxide for 10 min, target retrieval for 15 min (98-101°C), and protease plus for 30 min (40°C) (Advanced Cell Diagnostics, USA). A NiV-specific probe (Cat No. 439258, Advanced Cell Diagnostics) was incubated with the tissues for 2 h at 40°C. Amplification of the signal was performed using the RNAscope 2.5 HD Detection Kit – Red (Advanced Cell Diagnostics) according to the manufacturer’s instructions. Slides were digitally scanned and evaluated with the Nikon NIS-Ar software (Nikon, Praha, Czech Republic) to quantify the presence of viral RNA (percentage area positively stained).

All histopathological and ISH techniques were carried out in an ISO9001:2015 and GLP compliant laboratory and evaluation was performed by two qualified veterinary pathologists blinded to the treatment groups.

### 2.10 Statistical analysis

Statistical analyses were performed using Minitab, version 16.2.2. (Minitab Inc., USA) and GraphPad Prism, version 10 (GraphPad, UK). For comparison of survival, nonparametric distribution analysis (right censoring) was undertaken on Kaplan-Meier plots. A nonparametric Mann-Whitney’s *U* statistical test was applied to ascertain significance between groups. A significance level below 0.05 was considered statistically significant.

## 3 Results

### 3.1 Antibody responses to vaccination

**Figure 1:**
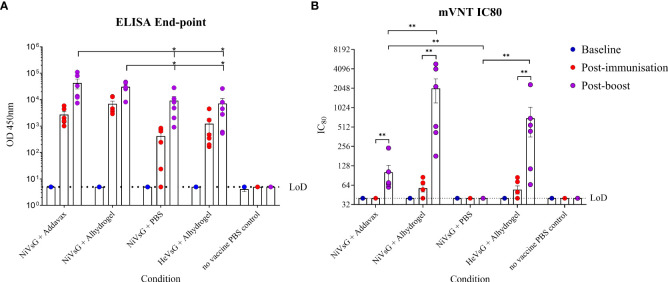
Analysis of sera taken from hamsters immunised with either NiV or HeV virus soluble G glycoprotein +/- adjuvant. Sera was sampled 1 day pre-immunisation, post-immunisation and post-boost. **(A)** Endpoint ELISA performed in duplicate using NiV-M antigen. **(B)** Neutralisation assay using a lentivirus based recombinant virus expressing NiV glycoproteins. Bars show mean values with error bars denoting standard error. Significance determined using the Mann-Whitney U test; *P<0.05 and **P<0.01. LoD, limit of detection.

Two soluble glycoprotein (sG) vaccine candidates, NiVsG and HeVsG, were evaluated alongside two different adjuvants: AddaVax™ and Alhydrogel^®^. All vaccine/adjuvant combinations elicited binding antibody responses by ELISA post-immunisation and post-boost (**Figure 1A**). Animals immunised with NiVsG without adjuvant showed lower antibody responses after single immunisation, but all animals demonstrated strong antibody binding responses post-boost, which interestingly, were comparable between the NiVsG without adjuvant group and the HeVsG plus Alhydrogel groups (**Figure 1A**). Animals immunised with NiVsG plus AddaVax or Alhydrogel had a significantly higher antibody response to those immunised with NiVsG without adjuvant (P=0.0411 and 0.0152, respectively) (**Figure 1A**).

Sera were also analysed by micro-virus neutralisation assay (mVNT) using a lentiviral based pseudovirus approach. A small amount of neutralising activity was observed after a single immunisation only amongst the groups with the Alhydrogel adjuvant; however, a significantly larger neutralising response was observed post-boost in all groups immunised with both NiV and HeV in the presence of either adjuvant (P<0.0087), suggesting a prime/boost schedule is necessary for induction of neutralising activity against NiV-M infection (**Figure 1B**). Alhydrogel induced a significantly increased neutralising response in animals immunised with NiVsG as compared to AddaVax (P<0.01), and without adjuvant there was no neutralisation activity observed despite the presence of binding antibodies (**Figure 1B**).

### 3.2 Post-challenge clinical parameters

**Figure 2:**
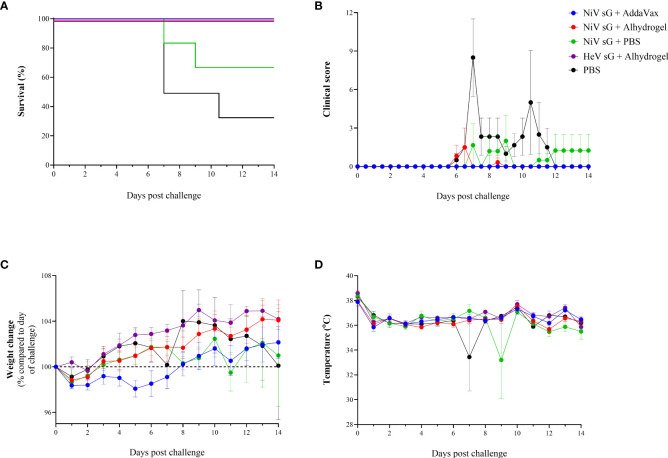
Clinical readouts from hamsters immunised with NiV or HeV virus soluble glycoprotein +/- adjuvant using a prime/boost strategy and challenged intraperitoneally with 100 TCID_50_ of NiV-M. **(A)** Kaplan-Meier survival plot. **(B)** Clinical observations. **(C)** Weight change compared to day of challenge. **(D)** Temperature. **(B–D)** Data show mean values with error bars denoting +/-standard error (n = 6/group).

Three weeks after receiving the homogenous boost immunisation, hamsters were challenged with 100 TCID_50_ NiV-M. In the unvaccinated group, 4/6 animals (67%) met humane clinical endpoint (HCE) by day 10 post-challenge. In those immunised with NiVsG without adjuvant, 2/6 (33%) animals met HCE. In contrast, all animals immunised with either NiVsG or HeVsG in the presence of adjuvant showed 100% survival post-NiV challenge (**Figure 2A**), a finding that was statistically significant compared to the unvaccinated group (P=0.018, Log-Rank survival analysis).

Clinical signs were observed in 5/6 unvaccinated animals, mainly consisting of ruffled fur, eyes closed, arched back, lethargy, laboured breathing and wasp-waisted (**Figure 2B**). In animals immunised with NiVsG without adjuvant, one hamster showed neurological manifestations and one animal showed signs similar to those in the unvaccinated group. In the NiVsG plus Alhydrogel group, one animal exhibited moderate clinical signs 6 days post-challenge - lethargy, ruffled fur, closed eyes, and arched back, which was resolved by the following day. No clinical signs were observed throughout the post-challenge phase of the study in animals immunised with NiVsG plus AddaVax and HeVsG plus Alhydrogel.

Following a slight decrease in weight directly after challenge, most of the animals showed a gradual increase thereafter. Hamsters immunised with NiVsG plus AddaVax exhibited a slight delay in weight gain compared to those immunised with the same antigen but with Alhydrogel adjuvant (**Figure 2C**). There was a noticeable decrease in weight in the groups where hamsters met HCE (NiVsG only and PBS control) which generally commenced ≤ 2 days before reaching HCE.

Temperatures remained relatively consistent for all animals, with outliers at timepoints where animals were found unresponsive in cage despite 6 hourly monitoring intervals being established (**Figure 2D**).

### 3.3 Viral RNA levels in peripheral blood and tissue

**Figure 3:**
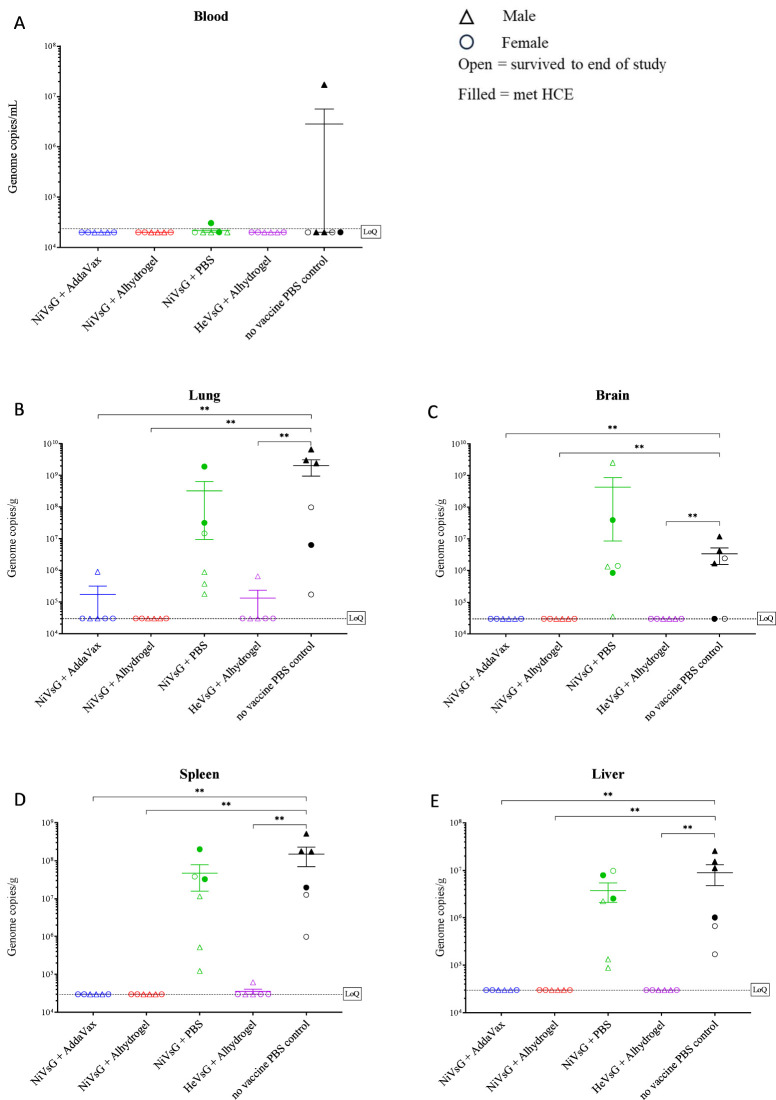
Quantitative RT-PCR analysis of viral genome copies per mL/g in whole blood and tissues of sG immunised hamsters after challenge with NiV-M. Samples collected from **(A)** blood, **(B)** lung, **(C)** brain, **(D)** spleen and **(E)** liver. Samples were collected when animals reached HCE or at end of study (day 14 post-challenge). Data points show values from individual animals with line and whisker plot denoting mean +/- standard error (n = 6/group); triangle = male, circle = female, open symbol = survived to end of study, filled symbol = met HCE. Dotted line = lower limit of detection/quantification (LoQ). Significance compared to PBS control group using the Mann-Whitney U test; **P<0.01.

Blood and tissue samples (lung, brain, spleen and liver) were taken upon reaching HCE or at the scheduled end of the study (14 days post-challenge), and the presence of viral RNA assessed using qRT-PCR. Viral RNA was detected in the blood of just one of the PBS control animals (1.77x10^7^ copies/mL) and at a much lower level (3.04x10^4^ copies/mL) in one of the hamsters immunised with NiVsG minus adjuvant (**Figure 3A**). No viral RNA was detected in the circulation of any of the animals immunised with sG plus adjuvant.

Viral RNA was detected in all but two of the tissues (the brain from two hamsters) in the unvaccinated control group (**Figures 3B–E**). Similarly, viral RNA levels were widespread in tissues from the group immunised with NiV without adjuvant, although only two animals from this group met HCE. In the group immunised with NiVsG plus AddaVax, viral RNA was detected in the lungs of one hamster but apart from this all other animals were negative. Similarly, in the group immunised with HeVsG plus Alhydrogel, viral RNA was detected in the spleen and lung from a single hamster. No viral RNA was detected in any of the tissues of the hamsters immunised with NiVsG plus Alhydrogel.

### 3.4 Histopathology

#### 3.4.1 Histopathological changes in tissues

**Figure 4:**
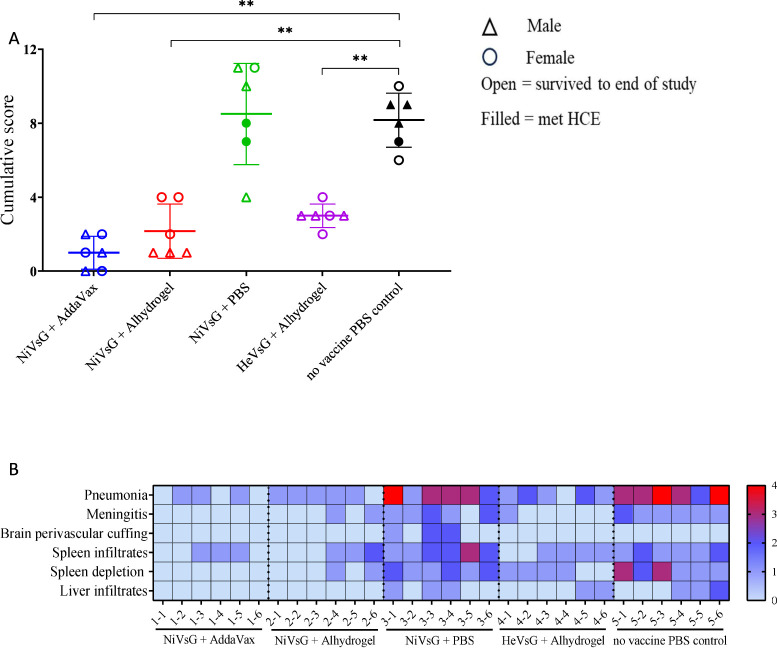
Histopathological results from sG immunised hamsters and control groups after challenge with NiV. **(A)** Cumulative histopathology scores of individual animals from the different experimental groups; triangle = male, circle = female, open symbol = survived to end of study, filled symbol = met HCE. Bars and whiskers represent mean ± S.D. Significance compared to PBS control group using the Mann-Whitney U test; **P<0.01. **(B)** Heatmap representation of the histopathological analysis. The severity of the lesions was recorded with a semi-quantitative score. The level of broncho-interstitial pneumonia in lung, the presence of meningitis and perivascular cuffing in brain, and the presence of infiltrates in spleen and liver, together with the degree of lymphoid depletion in the spleen were evaluated. For each parameter, the following scoring system was applied 0 = within normal limits; 1 = minimal; 2 = mild; 3 = moderate and 4 = marked/severe (n = 6/group).

Histopathological changes were mainly observed in organs from the PBS and NiVsG without adjuvant groups (**Figure 4A**). In the lung from these animals, a moderate to severe broncho-interstitial pneumonia was observed (score 3-4). However, the lungs from animals within the NiVsG (AddaVax), NiVsG (Alhydrogel) and HeVsG (Alhydrogel) groups showed a significant reduction in pathological findings compared to PBS control animals with only minimal to mild histopathological changes (score 0-2) (**Figure 4B**).

**Figure 5:**
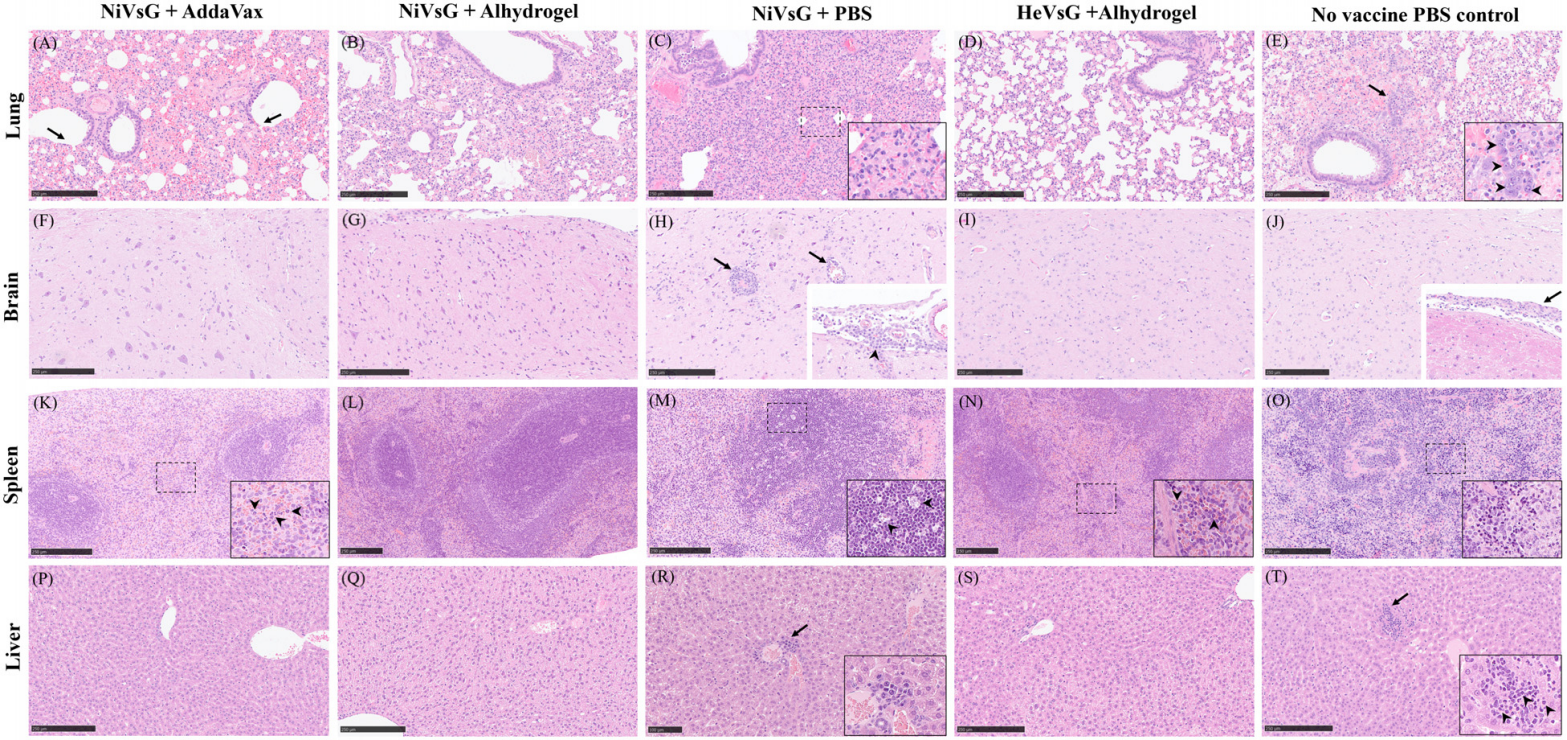
Representative histopathological images from the different experimental groups (H&E stain). **(A)** Lung section showing necrosis of the bronchiolar epithelium (arrows) and extravasated erythrocytes within the parenchyma. **(B)** Lung section showing mild interstitial pneumonia. **(C)** Lung section with severe interstitial pneumonia. Inset shows thickening of the alveolar walls (inflammatory mononuclear cell infiltration). **(D)** Lung section without histopathological changes. **(E)** Lung section with mild to moderate interstitial bronchopneumonia, oedema and type II pneumocyte hyperplasia (arrow and inset). **(F)** Brain section without histopathological changes. **(G)** Brain section without histopathological changes. **(H)** Brain section showing perivascular cuffing (arrows). Inset shows infiltrates of mononuclear inflammatory cells in the meninges (arrowhead). **(I)** Brain section without histopathological changes. **(J)** Brain section without histopathological changes. Inset shows thickening of the meninge with inflammatory cell infiltration (arrow). **(K)**. Spleen section showing mild inflammatory infiltration of polymorphonuclear cells within the red pulp. Inset and arrowheads show higher magnification of the polymorphonuclear cells. **(L)** Spleen section without histopathological changes. **(M)** Spleen section showing lymphoid depletion. Inset and arrowheads show tingible-body macrophages. **(N)** Spleen section showing minimal infiltration of polymorphonuclear cells and lymphoid depletion. Inset and arrowheads show the polymorphonuclear cells infiltration. **(O)** Spleen section with moderate splenic lymphoid depletion. Inset shows higher magnification of the lymphoid depletion area. **(P)** Liver section without histopathological changes. **(Q)** Liver section without histopathological changes. **(R)** Liver section showing small numbers of mononuclear inflammatory cells surrounding a central vein (arrow). Inset shows higher magnification. **(S)** Liver section without histopathological changes. **(T)** Liver section showing inflammatory infiltrate of mononuclear with occasional polymorphonuclear cells (arrow). Insert shows higher magnification. Arrowheads shows polymorphonuclear cells. Bar A-Q, S and T = 250 µm. Bar R = 100 µm.

Varying pathological changes were observed across the different tissues (**Figure 5**). Lung lesions consisted of multifocal to coalescing areas of broncho-interstitial pneumonia characterised by thickening of the alveolar walls (mainly mononuclear inflammatory cells) (**Figure 5C**, inset), type II pneumocyte hyperplasia (**Figure 5E**, inset) and necrosis of alveolar and bronchiolar epithelium. In the brain, meningitis showing mostly mononuclear cell infiltration was present in five out of six animals from the NiVsG group, and all six animals in the PBS group (**Figures 5H**, **J**, insets). Only two out of six animals from the NiVsG (Alhydrogel) group, and one out of six from the HeVsG (Alhydrogel) group showed minimal to mild meningitis (**Figure 4B**). In the brain sections of animals from the NiVsG group, perivascular inflammatory infiltrates (perivascular cuffing) were observed (**Figure 5H**, arrows). In the spleen, infiltration of polymorphonuclear cells and lymphoid depletion within the white pulp were mainly observed in animals from the NiVsG and PBS groups (**Figure 4B**) with all six animals in the group affected. In addition, tingible-body macrophages were occasionally observed (**Figure 5M**, arrowheads). In the liver, inflammatory cell infiltrates were composed mainly of mononuclear cells and occasional polymorphonuclear cells, mostly within the portal areas (**Figure 5T**).

#### 3.4.2 Virus RNA tissue distribution in tissues

**Figure 6:**
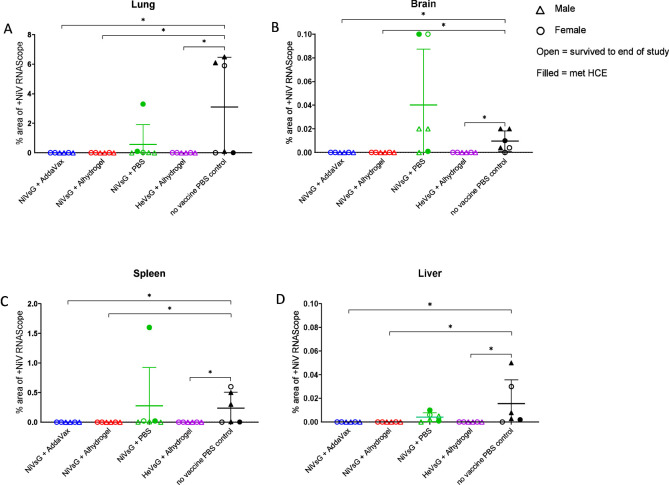
NiV RNA *in-situ* hybridisation (RNAScope) results from different experimental groups in **(A)** lung, **(B)** brain, **(C)** spleen and **(D)** liver. Data points show values (digital image analysis showing the percentage of positively stained area) from individual animals and lines denote mean ± S.D.; (n = 6/group); triangle = male, circle = female; open symbol = survived to end of study, filled symbol = met HCE. Significance compared to PBS control group using the Mann-Whitney U test; *P<0.05.

Viral RNA detected by ISH RNAscope was only observed in organs from the PBS and non-adjuvanted NiVsG groups, and not in any animals immunised with NiVsG or HeVsG in the presence of adjuvant (**Figure 6**). In the lung, the PBS group showed the higher RNA expression in comparison to those vaccinated with NiVsG, in which only one animal showed positive staining.

**Figure 7:**
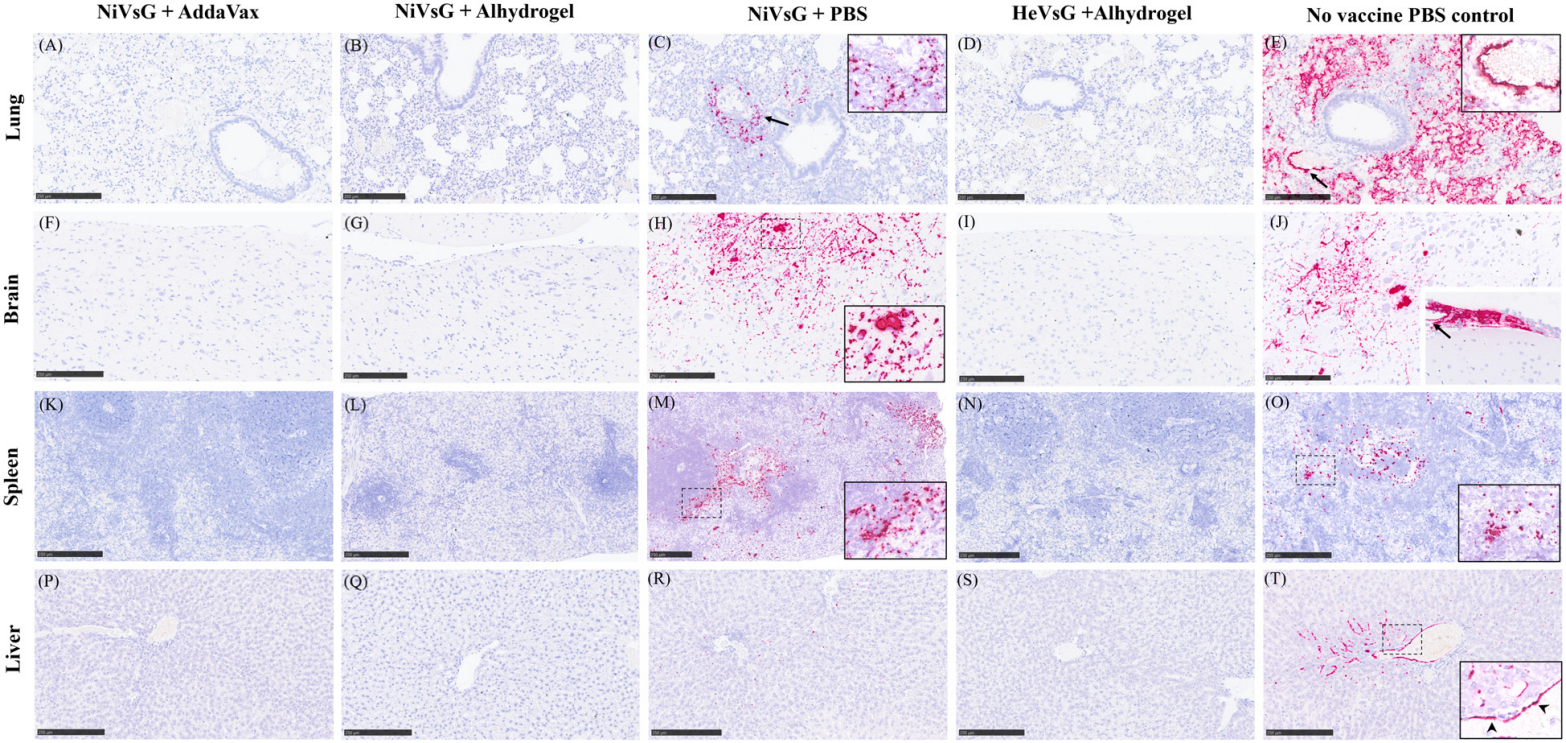
Representative images of NiV RNA *in-situ* hybridisation (RNAscope) staining in all experimental groups. **(A)** Lung section showing no viral RNA. **(B)** Lung section showing no viral RNA. **(C)** Lung showing viral RNA within the lung parenchyma around blood vessels and airways (arrow). Inset shows higher magnification. **(D)** Lung section showing no viral RNA viral. **(E)** Lung section showing large quantities of viral RNA within the lung parenchyma and endothelial cells from blood vessels (arrow). Inset shows higher magnification of viral RNA in endothelial cells from blood vessels. **(F)** Brain section showing no viral RNA. **(G)** Brain section with no viral RNA. **(H)** Brain section showing viral RNA in neuronal bodies and the neuropil within the midbrain. Inset shows higher magnification. **(I)** Brain section showing no viral RNA. **(J)** Brain section showing viral RNA in neuronal bodies and the neuropil of the midbrain. Inset shows thickened meninges showing large quantities of viral RNA within inflammatory cells and blood vessel endothelium (arrow). **(K)**. Spleen section showing no viral RNA. **(L)** Spleen section showing no viral RNA. **(M)** Spleen section showing viral RNA mostly within the red pulp. Inset shows higher magnification. **(N)** Spleen section showing no viral RNA. **(O)** Spleen section showing viral RNA within the white and red pulp. Inset shows higher magnification. **(P)** Liver section showing no viral RNA. **(Q)** Liver section showing no viral RNA. **(R)** Liver section showing small quantities of viral RNA within the liver sinusoids and occasional Kupffer cells. **(S)** Liver section showing no viral RNA. **(T)** Liver section showing viral RNA within the liver sinusoids, Kupffer cells, and endothelial cells from a central hepatic vein. Arrowheads in inset show viral RNA in endothelial cells. Bar A-L and N-T = 250 µm and M = 100 µm.

The levels and dissemination of viral RNA in tissues was analysed (**Figure 7**). In lung, viral RNA was observed in the areas of severe bronchopneumonia, especially in endothelial cells from the parenchymal capillaries (**Figures 7C, E**) and larger blood vessels (**Figure 7E**, arrow). In the brain, viral RNA was mainly detected in inflammatory cells within perivascular cuffs, neurons and the neuropil within the mid-brain regions and olfactory bulb (**Figures 7H, J**). Additionally, viral RNA was detected in meningeal inflammatory cell infiltrates and endothelial cells from meningeal blood vessels (**Figure 7J**, inset). In the spleen, viral RNA was observed throughout the parenchyma, mainly in the red pulp (**Figures 7M, O**). Finally, the presence of viral RNA in the liver was located within the liver sinusoids, Kupffer cells (**Figure 7T**), and endothelial cells from hepatic blood vessels (**Figure 7T**, arrow).

## 4 Discussion

In our study, we showed that a prime/boost immunisation schedule using the soluble glycoprotein (sG) from either Nipah virus (NiV) or Hendra virus (HeV) in conjunction with a commercially-available adjuvant (AddaVax™ or Alhydrogel^®^) elicited 100% survival in our hamster model of NiV infection. The efficacy of these sG is consistent with studies performed on other animal models, such as cats, ferrets, horses and African green monkeys (AGM) (27–29, 35, 36). In contrast, NiVsG immunisation without adjuvant led to a 33% of the group meeting humane clinical endpoints and presence of virus in multiple organs from these animals irrespective of survival.

A consistent increase in antibody levels was observed after both the prime and booster immunisation in all of the sG vaccinated groups, although some of the animals within the non-adjuvated group had very low/no response after prime only, demonstrating that both AddaVax and Alhydrogel supported antibody production after one dose. Further investigation is warranted to determine whether a single immunisation in the presence of these adjuvants would be sufficient to confer 100% survival. A single dose of HeVsG in the presence of Alhydrogel protected AGM after lethal challenge with either HeV and NiV, and similar results have been observed with other vaccine candidates including vesicular stomatitis virus (VSV)- and ChAdOx1-vectored immunogens, a VLP-based vaccine and a replicon particle system (37–41). Likewise, the addition of adjuvant significantly enhanced the immune response after the booster immunisation.

Low levels of neutralising antibody were detected in all groups after prime immunisation, similar to what was observed with CpG adjuvanted HeVsG in a challenge study in cats, and a non-adjuvanted vaccinia-vectored glycoprotein study in golden hamsters (34, 37, 42). These were significantly increased after boost in all the adjuvated groups, with no neutralising antibodies detected in the NiVsG alone or PBS control group. This suggests that adjuvant is necessary for the production of neutralising antibodies with this vaccine approach, with Alhydrogel inducing higher titres than AddaVax after boost. This is unsurprising in the case of Alhydrogel being an alum (aluminium hydroxide) based adjuvant which induces a Th2 response by improving the attraction and uptake of antigen by antigen-presenting cells (APC), and this superior response has been noted in other studies (39, 43). In contrast, AddaVax has been shown to induce a Th1 response through the stimulation of cytokine and chemokine production by macrophages and granulocytes, although it may also enhance the Th2 response through the recruitment and activation of APC (44–46).

Raised antibody levels were observed in both ELISA and mVNT after immunisation with the HeVsG, suggesting good cross-reactivity and cross-neutralisation from this vaccine, in line with results from other studies (26, 28, 37, 40, 47). Further investigation into this would be useful to see if this cross-protectivity extends to more distantly related members of the *Henipavirus/Parahenipavirus* genus to determine the possibility of a pan-henipavirus vaccine (48, 49). Although it has been demonstrated that henipavirus-neutralising antibodies were unable to neutralise Cedar virus, and Cedar virus antibodies raised in rabbits against the coding region for the N protein failed to neutralise HeV and NiV in Vero cells (8).

A noticeable difference in antibody levels was seen between ELISA and mVNT assay readouts, suggesting that predominantly non-neutralising antibodies were detected through ELISA after prime immunisation in all vaccinated groups, and after boost-immunisation the neutralising function become more apparent in those immunised in the presence of adjuvant. Interestingly, clinical manifestations post-challenge aligned with total antibody response rather than neutralising antibody titres. All animals in the NiVsG plus adjuvant groups survived and 67% of the hamsters survived in the NiVsG minus adjuvant group, despite very low titres of neutralising antibody being observed in the NiVsG plus AddaVax group and no neutralising antibodies detected in the minus adjuvant groups. A similar response was seen in AGM in a single-dose vaccine regime with HeVsG adjuvanted with aluminium hydroxide and challenged with NiV-B (37). Although relatively low serum-neutralising antibodies against NiV were detected at the time of challenge, all animals were protected against a lethal dose of NiV-B, with only 2/6 showing signs of clinical illness. Other antibody functions that have been assessed and correlate with protection against NiV disease in hamsters are antibody-dependent compliment deposition (ADCD) and antibody-dependent cellular phagocytosis (ADCP) (21). Interestingly, a strong antibody response capable of recruiting complement proteins and cellular phagocytosis against the nucleoprotein has been reported, whereas in contrast only a weak response was observed against the glycoprotein, the antigen of choice in our vaccine (21).

No clinical signs of disease were observed in any of the animals immunised with the NiVsG + AddaVax or the HeVsG + Alhydrogel, and only one hamster in the NiVsG + Alhydrogel group showed moderate signs at a single timepoint before making a full recovery the following day. In contrast, severe clinical signs were recorded in 5/6 of the animals in the PBS control, and 2/6 in the NiVsG group without adjuvant, with two showing signs of neurological sequalae and two experiencing laboured breathing. This parallels similar henipavirus sG plus adjuvant immunisation studies in cats, ferrets, AGM and hamsters, although for the latter, three doses of NiVsG were required for the prevention of clinical manifestations (27–29, 36, 42, 50, 51).

Of animals meeting HCE, two presented with neurological disease manifestations (one in the PBS control group and one from the unadjuvanted NiVsG group), a lower percentage than what has been recorded in other low-dose challenge studies (24, 52). This may be because these hamsters reached HCE through pulmonary complications, such as pneumonia before this could be manifested. Evidence of moderate to severe interstitial pneumonia in the histopathological analysis of these groups support this hypothesis. Viral RNA and histopathological changes (principally meningitis) were detected in the brain from most of the animals from both these groups, demonstrating neural involvement, indicating that if the study had lasted longer these may have manifested in neurological signs. Importantly, no viral RNA was detected in the brain of any of the adjuvanted vaccine groups.

Viral RNA was absent in all of the tissues from the group immunised with NiVsG plus Alhydrogel, and only relatively low levels (<1.0x10^6^ copies/g) were recorded across three of the animals (lung and spleen) in the other vaccine adjuvated groups. Due to practical constrains, the viability of the virus in these tissues was unable to be assessed. This is in marked contrast to the non-adjuvanted and control groups where high levels of viral RNA were recorded across a breadth of tissues. The low level of circulating viral RNA reflected that of our earlier study, where little-to-no virus was detected in the blood from intranasally or intraperitoneally challenged hamsters culled at days 2, 4 post-challenge, or upon reaching HCE (24). Rockx et al., 2011 and Baseler et al., 2016, also failed to detect infectious virus in the blood of their hamster models of henipavirus disease, although for the latter this was only up to 48 hrs post-challenge, supporting the idea that initial viral dispersal may be by means other than the hematogenous route (52–54).

Severe pneumonia and spleen depletion/infiltrates was observed in all the non-adjuvanted and PBS controls, whereas relatively few histopathological changes were recorded in tissues from the vaccine + adjuvant groups, mainly marked by mild pneumonia. Meningitis was observed in 11/12 of the non-adjuvanted/PBS control groups but only 3/18 of the vaccine + adjuvant groups. No meningitis or brain perivascular cuffing was observed in the animals vaccinated with NiVsG +AddaVax.

Results from RNA *in-situ* hybridisation (RNAScope) aligned closely with the qRT-PCR analysis, albeit at a lower level. This reduction in sensitivity meant that RNA expression was only observed in the NiVsG minus adjuvant and PBS control groups.

Several limitations would need to be addressed in future studies. We did not test groups containing HeVsG plus AddaVax or minus adjuvant, or adjuvant only controls due to the logistical challenges of working at CL4 limiting the number of animals we could use. Other studies have shown that adjuvant only controls have provided a level of protection against NiV challenge in the absence of any specific antigen (30, 39). Also, male vs female cohorts were too small (n=3) to support statistical analysis of differences between the sexes. Future studies would also benefit from investigation into cell-mediated responses associated with henipavirus infection, although the supply of hamster specific immunological reagents is currently limited (22, 55).

## 5 Conclusions

In this study we demonstrate that vaccination with NiVsG in the presence of two commercially available adjuvants supported 100% survival in hamsters after challenge with NiV-M, and that this protection was also conferred after vaccination with the sG from another species of henipavirus in the presence of adjuvant. The establishment of a validated hamster model of henipavirus disease within the UK enhances capacity for evaluating candidate vaccines and therapeutics against this priority pathogen.

This research further supports to the practicality and value of developing a pan-henipavirus vaccine, important as a frontline intervention in the event of an outbreak from an unknown henipavirus. Future studies are necessary to ascertain the extent of this cross-protectivity, with the recent increase in discovery of novel and more distantly related parahenipaviruses in different species and in new locations around the world. This study also showed that substantial levels of neutralising antibodies were only observed after boost immunisation with the soluble glycoprotein in the presence of an adjuvant. However, these neutralising antibodies were not solely necessary for increasing survival rates or reducing clinical signs in the hamsters. More work is essential to understand the main drivers of vaccine-induced protection against henipavirus infection.

## Data Availability

The raw data supporting the conclusions of this article will be made available by the authors, without undue reservation.
